# Yiqi-Bushen-Tiaozhi Recipe Attenuated High-Fat and High-Fructose Diet Induced Nonalcoholic Steatohepatitis in Mice *via* Gut Microbiota

**DOI:** 10.3389/fcimb.2022.824597

**Published:** 2022-04-22

**Authors:** Junbin Yan, Yunmeng Nie, Yuan Liu, Jingya Li, Liyan Wu, Zhiyun Chen, Beihui He

**Affiliations:** ^1^ The First Affiliated Hospital of Zhejiang Chinese Medical University, Hangzhou, China; ^2^ The Second Central Laboratory, Key Lab of Integrative Chinese and Western Medicine for the Diagnosis and Treatment of Circulatory Diseases of Zhejiang Province, The First Affiliated Hospital of Zhejiang Chinese Medical University, Hangzhou, China; ^3^ Department of Gastroenterology, Tongde Hospital of Zhejiang Province, Hangzhou, China

**Keywords:** nonalcoholic steatohepatitis, Yiqi-Bushen-Tiaozhi recipe, Traditional Chinese Medicine, gut microbiota, α-Linolenic acidlipid metabolism

## Abstract

**Aim:**

To investigate the treating effect of Yiqi-Bushen-Tiaozhi (YBT) recipe on nonalcoholic steatohepatitis (NASH) mice, determine whether the outcome was associated with gut microbiota, and clarify the regulating mechanism.

**Methods:**

NASH mice were induced by high-fat and high-fructose diets (HFFD). In the fifth week, mice in the YBT group were orally administrated YBT (22.12g·kg^-1^·d^-1^) daily for 12 weeks. Fresh stool of mice was collected at the 16^th^ week for fecal 16S rDNA analysis. Hepatic pathology and biochemical indicators were used to reflect the improvement of YBT on hepatic inflammation and lipid metabolism in NASH mice. Quantitative real-time PCR (qRT-PCR) was used to verify the results of PICRUSt analysis.

**Results:**

Results of the pathological and biochemical index showed that YBT could improve NASH mice. Compared with improving inflammation and hepatocyte damage, YBT may be more focused on enhancing metabolic disorders in mice, such as increasing HDL-c level. The diversity and richness of the gut microbiota of NASH mice induced by HFFD are significantly different from the normal control (NC) group. After YBT treatment, the diversity and richness of the mice microbiota will be increased to similar NC mice. *Intestinimonas, Acetatifactor, Alistipes, Intestinimonas, Acetatifactor*, and *Alistipes* have the most significant changes in the class level. PICRUSt analysis was performed to predict genomic functions based on the 16S rDNA results and reference sequencing. The efficacy of YBT in the treatment of NASH can be achieved by regulating the diversity and richness of gut microbiota. PICRUSt analysis results showed that the most relevant function of the microbiota construction variations is α- Linolenic acid (ALA) metabolism. Results of qRT-PCR showed significant differences between groups in the expression of Fatty acid desaturase 1 (FADS1), Fatty acid desaturase 2 (FADS2), Acyl-CoA Oxidase 1 (ACOX1), and Acyl-CoA Oxidase 2 (ACOX2) related to ALA metabolism. The expression of the above genes will be inhibited in the liver and small intestine of the HFFD group mice, and the expression can be restored after YBT treatment.

**Conclusion:**

YBT could treat NASH mice by improving the diversity and richness of gut microbiota and further the improvement of ALA metabolism.

## 1 Introduction

The prevalence of nonalcoholic fatty liver disease (NAFLD), which ranges from simple steatosis to its progressive form nonalcoholic steatohepatitis (NASH) and further fibrosis, cirrhosis, has been rising in the last decades (Gruneau et al., 2021), with 20-30% incidence rate in Western countries and 15-20% in Asia (Ashtari et al., 2015). Among obese patients, the prevalence of NAFLD/NASH even rises to as high as 80% (Younossi et al., 2019). Child fatty liver is also gradually becoming a severe public health problem (Hegarty et al., 2021). In addition, NAFLD/NASH replaces viral hepatitis as the leading cause of hepatocellular carcinoma (HCC) and liver transplantation (Terry Cheuk-Fung et al., 2021; Younossi et al., 2021). As the hepatic symptom of metabolic syndrome (MS), NAFLD/NASH is a risk factor for hypertension, diabetes mellitus (DM), and cardiovascular diseases (CVDs), and easily cooperates with the disorders mentioned to cause more severe outcomes (Baffy and Bosch, 2021; Kramer et al., 2021; Wu et al., 2021). Despite the increased morbidity and rigorous clinical impact, the pathogenic mechanisms of NAFLD/NASH are still unclear, and the specific treating methods are lacking (Cook et al., 2019). Therefore, it is critical to clarify the pathogenetic mechanism of NAFLD/NASH and select the appropriate therapeutic medicine in a targeted manner.

With the deepening of research, increasing evidence suggested that the gut microbiota participated in the progress of NAFLD/NASH (Ji et al., 2019) and is becoming the emerging target for treatment (Leung et al., 2016). The importance of Firmicutes and Bacteroidetes, microbial families, has been evaluated. An increased ratio of Firmicutes/Bacteroidetes is seen in over-weight or NAFLD patients, which tend to reduce with weight loss or alleviation of NAFLD, suggesting the richness of gut microbiota is associated with NAFLD progression (Boursier et al., 2016). The dysbiosis of microbiota diversity richness will induce the imbalance of lipid metabolism (Jin et al., 2021). The generation of beneficial lipids, polyunsaturated fatty acids (PUFAs), and high-density lipoprotein cholesterol (HDL-c) reduce. The level of detrimental substances such as saturated fatty acids (SFAs) and low-density lipoprotein cholesterol (LDL-c) increase, promoting NASH deterioration. Moreover, intestinal microbiota disorder is also a crucial intermediate mechanism associated with the abnormal accumulation of lipids in the liver and inflammation caused by chemical compounds (Ma et al., 2022; Zhang et al., 2022).

Traditional Chinese medicine (TCM) has been treasured by China and neighboring Asian countries for thousands of years as a conventional alternative treating method. TCMs are composed of multiple Chinese herbs, leading to the unique advantage that a formula could treat diseases *via* various mechanisms, and therefore attract attention worldwide. Yiqi-Bushen-Tiaozhi (YBT) recipe is composed of *Astragalus mongholicus bunge* (Huangqi), *Epimedium brevicornu Maxim* (Yinyanghuo), *Poria cocos(Schw.)Wolf* (Fuling), *Atractylodes macrocephala Koidz* (Baizhu), *Fallopia multiflora (Thunb.) Haraldson* (Heshouwu), *Crataegus pinnatifida Bunge* (Shanzha), *Sargassum pallidum (Turn.) C.Ag* (Haizao), *Curcuma aeruginosa Roxb* (Yujin), *Prunus persica (L.)* and *Batsch* (Taoren). YBT is a formula commonly used for the clinical treatment of NAFLD/NASH in The First Affiliated Hospital of Zhejiang Chinese Medical University, with an excellent curative effect in reducing lipid accumulation and losing weight in NAFLD/NASH patients. As the first line of nutrition intake, such as lipids, the intestinal microbiota is undoubtedly a crucial disease mechanism related to abnormal diets induced by NAFLD/NASH. Zhao S et al. have confirmed that ingested fructose affects hepatic fat accumulation and NAFLD/NASH progression through the gut microbiota (Zhao et al., 2020). Simultaneously, increasing evidence has implicated that TCMs may achieve the therapeutic effect *via* the gut microbiota (Zhao et al., 2019; Zhu et al., 2019). Therefore, in this study, we tried to study whether YBT can regulate the diversity and richness of intestinal microbiota and whether this is one of the multiple therapeutic mechanisms for YBT to improve NAFLD/NASH mice.

## 2 Materials and Methods

### 2.1 YBT Preparation

YBT was prepared by the Pharmacy Department of The First Affiliated Hospital of Zhejiang Chinese Medical University (Hangzhou, China), according to the guidelines of the Chinese Pharmacopoeia (Chinese Pharmacopoeia Commission China Medical Science Press: Beijing, 2015). The recipe consisted of the following herbs: Huangqi (purchased from Huadong Medicine Co., Ltd.), Yinyanghuo (Jun Tong Pharmaceutical Co., Ltd.), Fuling (Huadong Medicine Co., Ltd.), Baizhu (Hangzhou Tiandao Pharmaceutical Co., Ltd.), Heshouwu (Huadong Medicine Co., Ltd.), Shanzha (Hangzhou Xiaoshan Pharmaceutical Co., Ltd.), Haizao (Jun Tong Pharmaceutical Co., Ltd.), Yujin (Huadong Medicine Co., Ltd.), and Taoren (Hangzhou Xiaoshan Pharmaceutical Co., Ltd.) according to the ratio of 10:4:5:4:3:8:3:3:3. (w/w/w/w) **(** **Table 1** **).**

**Table 1:** Characteristics of the nine herbs in Yiqi-Bushen-Tiaozhi (YBT) recipe.

Chinese name	Botanical ID^a^	Botanical name^a^	Genus Family^a^		Used part	Weight (g)	Region
Huangqi	161231	Astragalus mongholicus bunge.	Leguminosae	Dried root		30	Inner Mongolia
Yinyanghuo	161102	Epimedium brevicornu Maxim.	Berberidaceae	Dried rhizome		12	Shanxi
Fuling	161203	Poria cocos(Schw.)Wolf	Polyporaceae	Dried sclerotium		15	Zhejiang
Baizhu	161208	Atractylodes macrocephala Koidz.	Compositae	Dried rhizome		12	Zhejiang
Heshouwu	170216	Fallopia multiflora (Thunb.) Haraldson	Polygonaceae	Dried root		10	Zhejiang
Shanzha	161130	Crataegus pinnatifida Bunge	Rosaceae	Mature fruit		24	Shandong
Haizao	170205	Sargassum pallidum (Turn.) C.Ag.	Sargassaceae	Dried frond		10	Shandong
Yujin	161206	Curcuma aeruginosa Roxb.	Zingiberaceae	Dried root		10	Sichuan
Taoren	161006	Prunus persica (L.) Batsch	Rosaceae	Mature seed		10	Shandong

a The herb name has been checked by The Plant List (http://www.theplantlist.org), Chinese Pharmacopoeia (http://db.ouryao.com/yd2015/), and Medicinal Plant Names Services (https://www.kew.org/science/our-science/science-services/medicinal-plant-names-services).

### 2.2 UHPLC-MS/MS Analysis

Our research group has identified the bioactive ingredients in YBT by Ultra-high performance liquid chromatography-MS/MS (UHPLC-MS/MS) (Rathod et al., 2019). 171 ingredients were screened out, among which 15.2% of the bioactive ingredients were carboxylic acids and derivatives, 12.9% were flavonoids, 12.3% were organ oxygen, 7.6% were isoflavones, and 6.4% were fatty acyls. In addition, 33.9% were classified into other courses, and 11.7% were not classified (Hong et al., 2020).

### 2.3 Animal Treating Processes

#### 2.3.1 Animals

Male C3H mice (7 weeks old, 16-18 gram) were purchased from Beijing Vital River Laboratory Animal Technology Co., Ltd. (Beijing, China). All experimental procedures followed the guidelines of The Animal Ethics Committee of Zhejiang Chinese Medical University (Approval lot: ZSLL-2016-138) and The National Guidelines for Experimental Animal Welfare.

#### 2.3.2 Modeling Processes

C3H mice were maintained on a 12h light/dark cycle at 22 ± 2°C with ad libitum access to a standard chow diet or a high-fat and high-fructose diet(HFFD)(36.1% fat, 21.6% protein, and 42.3% carbohydrate; supplied by Trophic Animal Feed High-tech Co., Ltd. and Nantong Trofi Feed Technology Co., Ltd. respectively) for 16 weeks. The NASH mice modeling method referred to the literature (Ibrahim et al., 2016; Van Herck et al., 2017). In the study, mice were randomly divided into three groups (n=5): Normal diet control (NC) group, fed with a standard chow diet and water; HFFD group, provided with the HFFD and 20% fructose water; YBT intervention group (HFFD-Y), fed with the HFFD in addition to daily administration of 26.76g·kg^-1^·d^-1^ YBT for treating from 5^th^ week. The reason for the dose of YBT is as follows. A total of 133g per serving of YBT. According to the standard adult weight (70kg), the dosage for adults was calculated as 1.90 g·kg^-1^. Next, mice were treated at 14 times (high dose) of the adult dose. Finally, 26.76g·kg-1·d-1 was obtained as the treating dose in the study. All mice were sacrificed with the anesthetic at the end of the 16^th^ week after fasting for 12 hours. Serum and liver tissues were collected for biochemical criteria and histopathology detections. Fresh stool samples were collected for Fecal 16S rDNA analysis. The remaining hepatic tissues and serum were stored at -80°C.

### 2.4 Pathological and Serum Biochemical Criteria Measurement

Hepatic tissues of mice were fixed with 10% neutral formaldehyde, dehydrated with ethanol, and embedded in paraffin. Then, the tissue was cut into five μm thick slices for detecting hepatic steatosis and inflammation *via* Hematoxylin and Eosin (H&E) and Masson staining.

The liver injury index (ALT/AST) and lipid metabolism index (TC/TG/HDL-c/LDL-c) in the serum of mice were detected with the corresponding kit and protocols. The analysis kits were both purchased from Diasys Diagnostic System GmbH (ALT lot:07398/00003352, AST lot: 07362/00003216, TG lot: 07407/00003293, TC lot: 07421/00003464, HDL-c lot: 22007/00003186, LDL-c lot: 23074/00003611). Hitachi 7020 automatic biochemical analyzer was used for detection.

### 2.5 Analysis of Fecal 16S rDNA

The database and software used in this analysis are shown in **Table 2**.

**Table 2:** The used databases and software in the study.

Name	Website
Databases	
The Plant List	http://www.theplantlist.org/
Chinese Pharmacopoeia	http://db.ouryao.com/yd2015/
Medicinal Plant Names Services	https://www.kew.org/science/our-science/science-services/medicinal-plant-names-services
UNITE	https://unite.ut.ee/
SILVA	https://www.arb-silva.de/
QIIME	http://qiime.org/
Software	
Trim Galore	https://github.com/FelixKrueger/TrimGalore
FLASH2	https://www.dgtech.com/flash2/
Mothur	http://mothur.org/
Usearch	http://www.drive5.com/usearch/
R project	https://www.r-project.org/
SPSS 22.0	https://www.ibm.com/
Metastats	http://metastats.net/
Lefse	https://huttenhower.sph.harvard.edu/lefse/

#### 2.5.1 Stool Sample Processing and DNA Extraction

The fresh fecal samples were collected from mice of three groups at baseline/16^th^ week, frozen immediately in liquid nitrogen, and stored at -80°C for further analysis. According to the protocol, fecal genomic DNA was extracted from the fecal samples with the QIAamp^®^ DNA StoolMini Kit (Qiagen, Hilden, Germany). The concentration and purity of DNA were detected through the Nanodrop, and the integrity was determined and verified through 0.8% agarose gel electrophoresis.

#### 2.5.2 High-Throughput Sequencing

The V3-V4 hypervariable region of the 16Sr DNA was amplified using the bacterial genomic DNA as a template. The forward (5’-CCTACGGGNGGCWGCAG-3’) and the reverse (5’-GACTACHVGGGTATCTAATCC-3’) primers were designed, and each was independently amplified three times. After checking the final PCR products by gel electrophoresis, the products from the same samples were pooled. The pooled PCR product was used as a template, and the index PCR was performed by using index primers for adding the Illumina index to the library. The amplification products were checked using gel electrophoresis and were purified using the Agencourt AMPure XP Kit (Beckman Coulter, CA, USA). The purified products were then indexed in the 16S V3-V4 library. The library quality was assessed on the Qubit@2.0 Fluorometer (Thermo Scientific) and Agilent Bioanalyzer 2100 systems. Finally, the pooled library was sequenced on an Illumina MiSeq 250 Sequencer for generating 2×250 bp paired-end reads.

#### 2.5.3 Data Quality Control

In order to obtain high-quality sequencing data for improving subsequent bioinformatics analysis accuracy, quality control and filtering of the original data were needed. The central processing steps are as follows: i) Trim Galore was used to remove bases with an end mass of less than 20 and adapter sequences, followed by short sequences of less than 100 bp in length; ii) Fast Length Adjustment of Short reads (FLASH, v1.2.11) (Magoč and Salzberg, 2011) was used to splice the paired sequences obtained by double-terminal sequencing, and further remove the sequences that were still of low quality after merge (less than 20); iii) Mothur was used to find and remove primers in the sequences; iiii) Usearch was used to remove sequences with a base error rate greater than two or sequences with a length less than 100 bp. Clean reads with high quality and reliability were finally obtained for the subsequent analysis.

#### 2.5.4 Clustering and Annotation

##### 2.5.4.1 Operational Taxonomic Units Clustering

Operational Taxonomic Units (OTU) is an analysis method that calculates the similarity of sequences, setting a specific classification threshold (in the study, the threshold is similarity >97%) and obtaining the distance matrix with the same threshold value for clustering and classifying.

OTU analysis process in the study is as follows. i) Repetitive sequences whose length and base composition are precisely the same are deleted. Non-completely repeated sequences are extracted for subsequent analysis; ii) The sequence after de-duplication is sorted according to the number of repeats from largest to smallest; iii) UPARSE method (Edgar, 2013) is used to remove the sequence that appears only once in all samples (Singleton sequence); iiii) The sequences are clustered with 97% similarity. Sequences with a similarity greater than 97% will be grouped into the same OTU. Meanwhile, *de novo* clustering analysis removes the chimera sequence. The final OTU representative sequence will be used for subsequent species annotation.

##### 2.5.4.2 Gut Microbiota Annotation

UNITE (https://unite.ut.ee/) (Kõljalg et al., 2005) and SILVA (https://www.arb-silva.de/) (Quast et al., 2013) were crucial public databases used to search the information of species. Software Mothur was used to find the species information from the above databases with the highest similarity to OTU sequences (the confidence>80%) for gut microbiota annotation. After successfully annotating the microbiota, muscle and FastTree (Price et al., 2010) were used to construct the phylogenetic tree to reflect the evolutionary relationships of the gut microbiotas.

#### 2.5.5 Analysis of Difference in Colony Composition

Based on the OTU that has been annotated for the gut microbiota species, the R package ggplot2 was used to analyze the colony composition. According to class, family, and genus, we drew the gut microbiota pie charts of each group (selecting the microbiota with a relative abundance greater than 1%) to show the distribution of the gut microbiota.

Although the pie chart could directly reflect the ratio of the mouse gut microbiota, there are various microbiota classifications at the genus level, and the proportions are similar, so it is not suitable for using pie charts. Therefore, we decided to directly screen the microbiota (at the genus level) with significant differences in the ratio of mice in the NC, HFFD, and HFFD-Y groups. We used the software Metastats to compare the proportion of gut microbiota and find the microbiota with significant differences at the genus level. *P-value <*0.05 was seen as the threshold to screen for significant differences.

#### 2.5.6 Diversity and Richness Analysis of Mice Gut Microbiota

##### 2.5.6.1 Alpha Diversity Analysis

Alpha diversity refers to the analysis of samples to reflect the richness and diversity of the microbial community. In the study, Alpha diversity analysis can measure the abundance and diversity of the gut microbiota in each mouse. Software mothur was used for analysis. The index and formula for evaluating the richness and diversity are as follows:

I. Index reflecting the community richness of gut microbiota;

i) Observed_species: The number of directly observed OTUs.

ii) Chao1 (https://mothur.org/wiki/Chao/) is used to assess the total number of species in the sample. The higher the value, the more abundant the gut microbiota of the mice. The algorithm is as follows.


$$S_{chao1}=S_{obs}+\frac{n_{1}(n_{1}-1)}{2(n_{2}+1)}$$

S_chao1_: the estimated community richness; S_obs_: the observed number of OTUs; n_1_: the number of OTUs with only one sequence; n_2_: the number of OTUs with only two sequences.

iii) ACE (https://mothur.org/wiki/ace/) is also used to estimate the total number of species. The higher the value, the more abundant the gut microbiota of mice. But the algorithm is different from Chao1.


$$\{\begin{array}{c} \;\;\;S_{abund}+\frac{S_{rare}}{C_{ACE}}+\frac{n_{1}}{C_{ACE}}{\hat{\gamma }}^{2}ACE,for\;\gamma_{ACE}<0.80\\ \;\;\;S_{abund}+\frac{S_{rare}}{C_{ACE}}+\frac{n_{1}}{C_{ACE}}{\tilde{\gamma }}^{2}ACE,\;for\;\gamma_{ACE}\geq 0.80 \end{array}$$

S_rare_: the number of OTUs with ‘abund’ or fewer individuals; S_abund_: the number of OTUs with more than ‘abund’ individuals; n_i_: the number of individuals in the ith OUT; abund: the threshold of ‘abund’ individuals (the selecting threshold is 10 in the study).

II. Index reflecting the community diversity of gut microbiota;

i) Shannon (http://www.mothur.org/wiki/Shannon/) is used to estimate the diversity of species in samples. The higher the value of Shannon, the higher the diversity of the mouse gut microbiota. The algorithm is as follows.


$$H_{Shannon}=-\sum_{i=1}^{Sobs}\frac{n_{i}}{N}ln\frac{n_{i}}{N}$$

S_obs_: the number of observed OTUs; n_i_: the number of individuals in the i^th^ OUT; N: the total number of individuals in the community

ii) Simpson (https://mothur.org/wiki/simpson/) is also used to estimate the diversity of species in the sample. Simpson is negatively correlated with species diversity. The higher the index value, the lower the diversity of the microbiota.


$$D_{simpson}\frac{\sum_{i=1}^{Sobs}n_{i}(n_{i}-1)}{N(N-1)}$$

S_obs_: the number of observed OTUs; n_i_: the number of individuals in the i^th^ OUT; N: the total number of individuals in the community

Kruskal-Wallis rank sum test was used to determine whether there was a significant difference in Alpha diversity among the three groups of mice. *P-value <*0.05 was seen as the difference significance screening threshold. Bonferroni was used to perform multiple testing on the *p-value*.

##### 2.5.6.2 Beta Diversity Analysis

Beta diversity analysis refers to the abundance of sequencing results to calculate the distance between samples, reflecting differences in microbial communities. The content of Beta diversity analysis is as follows.

I. Venn diagram was used to show the intersection OTUs of the mouse gut microbiota in the NC, HFFD, and HFFD-Y groups.

II. Comparing the similarity between samples as a whole through the clustering tree. Based on the OTU abundance, the Bray-Cruits dissimilarity matrix between samples was calculated, then unweighted pair group method with arithmetic mean (UPGMA) clustering was performed. The mouse with similar gut microbiota will be clustered.

III. Principal Component Analysis (PCA) was used to reduce the dimensionality of OTU abundance (Wang et al., 2012). Variance decomposition was used to show the differences of gut microbiota on the three-dimensional coordinate map. The more similar the composition of the gut microbiota, the more mice clustered in the PCA chart.

IV. However, the above analysis only reflected the similarity/difference of the gut microbiota between/in groups of mice but cannot conclude whether the difference is statistically significant. Thus, ADONIS analysis, also known as Permutational Multivariate Analysis of Variance Using Distance Matrices (PERMANOVA) analysis based on distance or dissimilarity matrix, was used to decompose the overall variance between samples, analyze the interpretation degree of differences between grouping, and use permutation test to calculate whether the difference between groups is significant. Bray-curits dissimilarity matrix was selected, and 9999 permutations were performed on the group to which the sample belongs. In the results, R2 represents the degree of explanation of the grouping to the difference between the samples. *Pr <*0.05 indicates the result is statistically significant.

V. ADONIS analysis assesses whether there are significant differences in gut microbiota between groups as a whole but cannot screen out the specific microbiota causing these significant differences. Therefore, we further used Linear discriminant analysis Effect Size (LEFSe) to screen the microbiotas most likely to explain the differences between mice (Zhang et al., 2013). Lefse software was used for analysis. **i**)First, non-parametric Kruskal-Wallis rank sum test was used in multiple samples to screen microbiotas with significant differences in abundance; **ii**)Then paired Wilcoxon rank sum test was used to analyze the differences; **iii**)Finally, linear discriminant analysis (LDA) was used to evaluate the LDA score of selected microbiotas with a significant difference. Common logarithm conversion was used on the LDA score. The higher the absolute value of the LDA score, the more importance of the microbiota in causing the difference between the NC, HFFD, and HFFD-Y groups. We used |LDA|>2 and *p-value <*0.05 as the screening threshold.

#### 2.5.7 PICRUSt Predictive Analysis

Finally, we used the software PICRUSt to predict the function of the gut microbiota based on OTUs. The analysis steps are as follows: i) Predicting and constructing the gene composition and 16S copy number of all microbiota in the GreenGene database based on the annotation information of known relative species; ii) *Via* QIIME database, the OTU table was obtained based on the closed-reference algorithm. Then, using the above 16S copy number information to correct the OTU abundance (divide the OTU abundance by the copy value corresponding to the OTU) to obtain the corrected OTU table; iii) Combining the corresponding relationship between KEGG-based gene family and OTU, we converted the corrected OTU table of the gut microbiota of mice into a gene family abundance (function) table; iiii) The gene family abundance (function) table was further summarized to a higher functional classification level. We next respectively compared the functional differences of the gut microbiota between the NC/HFFD groups and HFFD/HFFD-Y groups *via* Welch’s t-test. A more stringent *p-value* (<0.01) was used as the screening threshold to ensure higher reliability of results.

### 2.6 qRT-PCR Verification

500ul lysis buffer, three grinding beads, and 20mg liver and intestine tissues were respectively added into the grinding tube and subsequently broken with a homogenizer (5000rpm, 20 seconds/5000rpm, 20 seconds, rep3). 300ul of supernatant was absorbed into a new 1.5mL RNase Free Tube. Next, RNA was extracted using the RNA-Quick Purification Kit to remove genomic DNA. The reverse reaction was performed with 15 mins at 37°C and 5 seconds at 85°C. Finally, 10 μl PCR reaction mix was built, including 2μl cDNA, 5μl 2xSuper SYBR green, 0.4μl forward prime (10μm), 0.4μl reverse primer (10μm), and 2.2μl ddH2O. RNA-Quick Purification Kit (cat: RN002plus; lot: 20210801), Fast All-in-One RT kit (cat: RT001; lot: 20211001), and 2xSuper SYBR Green qPCR Master Mix (cat: QP002; lot: 20211001) were purchased from ES Science.

By searching Kyoto Encyclopedia of Genes and Genomes (KEGG) database, we found that alpha-linolenic acid metabolism (map00592) was mainly related to the decomposition of ALA *in vivo*. To verify the relevant functions of the gut microbiota, we used qRT-PCR to detect the RNA expression of crucial enzymes in alpha-Linolenic acid metabolism. We selected Fatty acid desaturase 1 (FADS1), Fatty acid desaturase 2 (FADS2), Acyl-CoA Oxidase 1 (ACOX1), and Acyl-CoA Oxidase 2 (ACOX2) closely related to ALA metabolism in the alpha-Linolenic acid metabolism pathway. FADS1 and FADS2 are both hub enzymes in the metabolic pathway of ALA, which could promote the absorption and utilization of ALA *in vivo* (Koletzko et al., 2019). ACOX1 and ACOX2 are also enzymes closely involved in lipid metabolism, including ALA. Up-regulation of ACOX1 and ACOX2 could improve lipid accumulation and inflammation in the liver, thus treating NASH mice (Liu et al., 2019). In addition, these enzymes are not plant-specific ALA metabolic enzymes and could exist in animals (Homo sapiens, Rattus norvegicus, and Mus musculus) as well. Although the above genes are all enzymes closely related to ALA metabolism, they can only indirectly reflect the metabolism of ALA. Therefore, in order to increase the reliability of the conclusion, we not only verified the expression changes of the above genes in the liver of mice, but also verified the expression changes in the small intestine.

The information and primer sequences of the critical enzymes are listed in **Table 3**. Primer sequences were synthesized by Sangon Biotech. We designed primer sequences with reference to the NCBI database (https://www.ncbi.nlm.nih.gov) and PrimerBank (https://pga.mgh.harvard.edu/primerbank/). Actin was selected as the reference gene. 2-^△△^Ct was used for calculation. Each sample was repeated three times, and the average data value was used.

**Table 3:** mRNA primer sequences.

Gene	Description	Gene id	Primers (5’→3’)
Actin	actin	11461	Forward GGGAAATCGTGCGTGACA
			Reverse CAAGAAGGAAGGCTGGAAAA
FADS1	fatty acid desaturase 1	76267	Forward AGCACATGCCATACAACCATC
			Reverse TTTCCGCTGAACCACAAAATAGA
FADS2	fatty acid desaturase 2	56473	Forward AAGGGAGGTAACCAGGGAGAG
			Reverse CCGCTGGGACCATTTGGTAA
ACOX1	acyl-CoA oxidase 1	11430	Forward TAACTTCCTCACTCGAAGCCA
			Reverse AGTTCCATGACCCATCTCTGTC
ACOX2	acyl-CoA oxidase 2	93732	Forward CACCCTGACATAGACAGTGAAAG
			Reverse CTGGGTCACGTTGGATGAGG

### 2.7 Statistical Analysis

All statistical analyses were performed with SPSS 22.0 and GraphPad Prism 8. Data were expressed as the means±SEM (standard error of mean). One-way analysis of variance (ANOVA) was used to compare differences between two groups. Kruskal Wallis test was performed when more than two groups were present. Differences were considered statistically significant at the *p-value <*0.05.

## 3 Results

### 3.1 Results of Pathologology and Serum Biochemical Criteria

**Figure 1:**
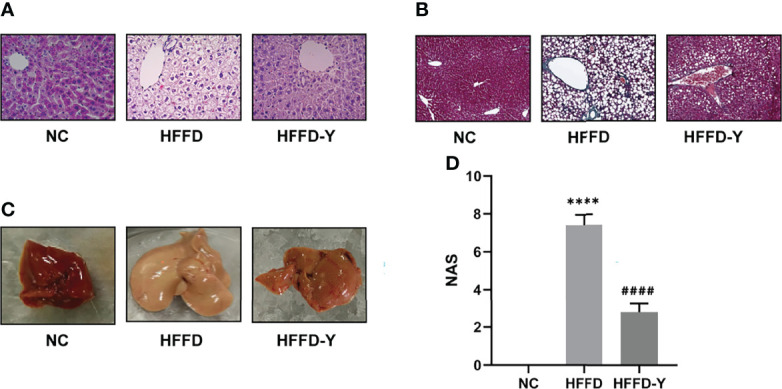
Pathological results of mice. **(A)** H&E staining results of the mice. **(B)** Masson staining results of the mice. **(C)** The photos of the mice’s liver. **(D)** The NAS calculation results of the mice. (^****^ *p*<0.0001 vs NC; ^####^ *p*<0.0001 vs HFFD).

H&E and Masson staining was performed on the liver tissues of mice in NC, HFFD, and HFFD-Y groups to analyze the hepatic histology for determining the results of NASH mice modeling and the improvement of hepatic inflammation and steatosis by YBT. Compared with the hepatic tissues from the NC mice, the tissues from mice in the HFFD group showed more extensive and robust steatosis accompanied by intralobular inflammatory foci. After the intervention of YBT, hepatic inflammation and steatosis in the mice of the HFFD-Y group were improved compared with the HFFD group mice (**Figure 1A**). The hepatocytes of the mice in the NC group were neatly arranged, and there were a few blue collagen fibers in the portal area. In contrast, the staining of collagen fibers in the HFFD group was significantly increased, and some fibrous septa were formed but without the formation of typical hepatic pseudo lobules. The collagen fibers in the liver of mice after YBT treatment were reduced to varying degrees (**Figure 1B**). From the photos of the liver of the mice, we can also find that the livers of the mice in the HFFD group were significantly whiter and larger than the livers of the NC and HFFD-Y mice, suggesting that the livers of the HFFD mice may have accumulated more lipids (**Figure 1C**). In addition, the results of NAFLD activity score (NAS), which is a semi-quantitative data used to assess NAFLD progression (NAS >4 is considered to have progressed to NASH) (Brunt et al., 2011), also suggested that HFFD could induce mice liver to show the disease characteristics of NASH (hepatocyte steatosis with inflammation), supporting the success of HFFD-induced NASH mice. YBT improved the steatosis and inflammation of NASH mice liver (significantly lower NAS score, *p-value <*0.0001), which supports the conclusion that YBT could treat NASH mice (**Figure 1D**). The above pathological results all support the success of modeling in the study, and that YBT has the effect of improving the lipid accumulation of inflammation of mice liver.

**Figure 2:**
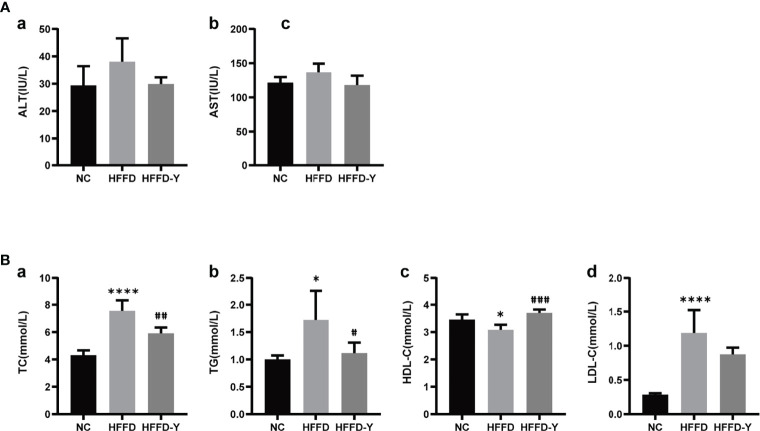
Results of serum biochemical criteria detection. **(A)** Results of hepatic injury indicators. **(B)** Results of lipid level. (*p<0.05 vs NC, ****p<0.0001 vs NC; ^#^ *p*<0.05 vs HFFD, ^##^ *p*<0.01 vs HFFD, ^###^ *p*<0.001 vs HFFD).

When the liver is damaged, the Alanine aminotransferase (ALT) and Aspartate aminotransferase (AST) in hepatocytes will enter the blood, leading to increased serum ALT and AST levels, suggesting the occurrence of liver disease. Therefore, serum ALT and AST levels are directly proportional to liver damage and are the most commonly used clinical liver function test indicators. The liver injury indicators results showed that the HFFD diet could cause a certain degree of liver injury in mice, and the hepatic injury would be improved after YBT intervention. However, the results are not statistically significant (**Figure 2A**).

Triglyceride (TG), total cholesterol (TC), high-density lipoprotein cholesterol (HDL-c), and low-density lipoprotein cholesterol (LDL-c) are clinical indicators used to reflect blood lipids and lipid metabolism. TC refers to the sum of cholesterol in the blood. HDL-c is the lipoprotein with the highest density and the smallest particles in the blood. It is responsible for transporting cholesterol in the plaque to the liver for catabolism, that is, reverse cholesterol transport, reducing the deposition of cholesterol on the blood vessel wall, enacting an anti-arteriosclerotic effect. LDL-c is responsible for transporting cholesterol from the liver to the plaque. Increased LDL-C is the leading risk factor for the occurrence and development of atherosclerosis. **Figure 2B** shows that the HFFD diet could significantly increase the serum levels of TC, TG, and LDL-c and reduce HDL-c (*p-value <*0.05). After YBT treatment, the above blood lipid indexes will be significantly improved (*p-value <*0.05). Among them, YBT treatment has the best improvement effect on regulating serum HDL-c levels(*p-value <*0.001).

Results of serum biochemical criteria detections suggested that the crucial mechanism of HFFD diet-induced NASH mice may be the inducing disorder of lipid metabolism, but not hepatic injury. Additionally, lipid metabolism is also an essential regulatory mechanism for YBT to treat NASH mice. The level of mice serum HDL-c may be the primary object for YBT to improve lipid metabolism in mice.

### 3.2 Results of Fecal 16S rDNA Analysis and Bioinformatics

#### 3.2.1 Results of Data Control

A total of 644,541 high-quality clean reads were obtained from 15 stool samples of mice for subsequent bioinformatics analysis. Details are shown in **Table 4**.

**Table 4:** Sequencing data control results.

Sample	Raw reads	Q20(%)^a^	Q30(%)^a^	Clean reads	Clearance(%)	Q20(%)^b^	Q30(%)^b^
NC1	50963	97.41	95	44489	12.7	98.92	97.65
NC2	42772	97.49	95.12	37398	12.6	98.91	97.62
NC3	61282	97.45	95.06	53638	12.5	98.94	97.66
NC4	54857	97.47	95.08	48129	12.3	98.93	97.64
NC5	48538	97.45	95	42467	12.5	98.91	97.62
HFFD1	50924	97.35	94.88	43974	13.6	98.9	97.65
HFFD2	48476	97.4	94.97	42165	13.0	98.88	97.61
HFFD3	41431	97.55	95.23	36101	12.9	98.94	97.72
HFFD4	62077	97.44	95.03	53476	13.9	98.87	97.57
HFFD5	50172	97.34	94.88	43684	12.9	98.9	97.6
HFFD-Y1	48949	97.42	95.01	42587	13.0	98.9	97.59
HFFD-Y2	52449	97.52	95.18	45909	12.5	98.91	97.63
HFFD-Y3	43235	97.24	94.73	37811	12.5	98.9	97.59
HFFD-Y4	30244	97.33	94.83	26157	13.5	98.86	97.52
HFFD-Y5	53039	97.42	95.03	46556	12.2	98.9	97.64

^a^Quality control results of Raw reads; ^b^Quality control results of Clean reads.

Millions of reads will be produced after being sequenced. The sequence data transformed by Base Calling from the raw image obtained by sequencing are Raw reads. Clean reads are the high-quality Raw reads that have been processed. Clearance reflects the percentage of Raw reads removed because of low quality. Q20 and Q30 are the percentages of the quality value of a certain base in the total number of bases, which could reflect the quality of sequencing data. The higher the value, the better the quality of sequencing data. The value of Q20 and Q30 higher than 85%, indicating the data quality is qualified.

#### 3.2.2 Results of Classification and Annotation

In total, 678 OTUs were collected. Of these, only 241 OTUs were successfully annotated as the relevant gut microbiota. We selected the top 100 OTUs with the highest abundance to draw the evolutionary tree (**Figure S1**). The phylogenetic tree could indicate the evolutionary relationship between species that are considered to have a common ancestor. Each node in the tree represents the nearest common ancestor of each branch, and the length of the line segment between nodes corresponds to the evolution distance. The OTUs on the same branch indicate a close evolutionary relationship.

#### 3.2.3 Gut Microbiota Variations in Response to HFFD and YBT Intervention

**Figure 3:**
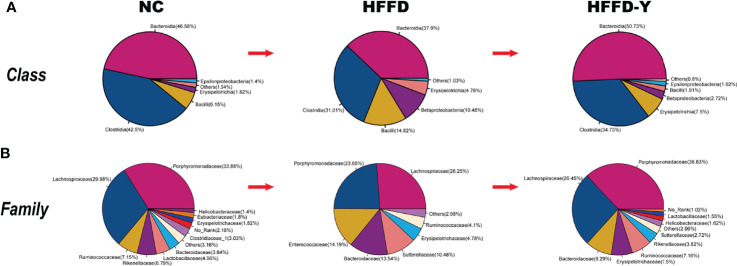
Gut microbiota proportion and variations. **(A)** Analysis results at the class level. **(B)** Analysis results at the family level.

Analysis of differential gut microbiota among the NC, HFFD, and HFFD-Y groups showed that the most abundant species of gut microbiota (more than 80% in total) at the class level were *Bacteroidia*, *Clostridia*, and *Bacilli* (**Figure 3A**). Compared with the mice of the NC group, the proportion of *Bacteroidia* and *Clostridia* in HFFD-induced NASH mice will decrease, and the intervention of YBT will restore the proportions. In the mice of the HFFD group, the ratio of *Bacilli* was higher than that of the NC group and decreased after YBT treatment. At the family level, the dominant gut microbiota is *Porphyromonadaceae* and *Lachnospiraceae* (more than 50% in total). HFFD reduced the proportion of *Porphyromonadaceae* and *Lachnospiraceae* in the intestines of mice, while YBT treatment could recover them (**Figure 3B**).

**Figure 4:**
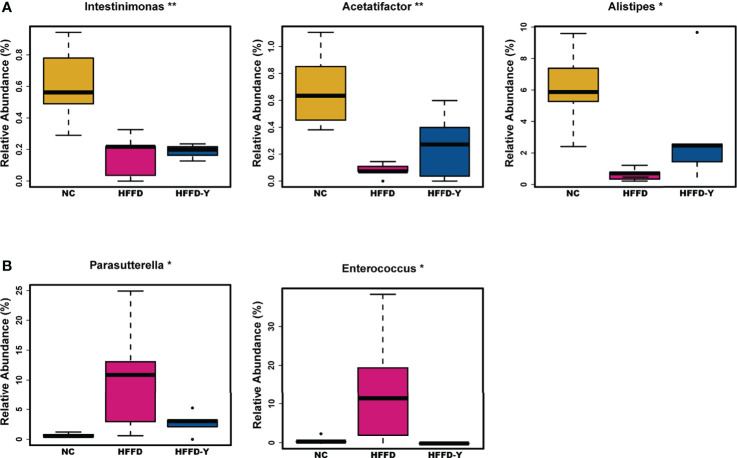
Gut microbiota variations at the genus level. **(A)** Microbiota with the abundance descend first and then ascend. **(B)** Microbiota with the abundance ascend first and then descend. (*p<0.05 vs HFFD, **p<0.01 vs HFFD).

At the genus level, due to the variety of gut microbiota and similar proportions, we focused on screening the microbiota communities with significant variations. The proportion of *Intestinimonas*, *Acetatifactor*, and *Alistipes* in the mice of the HFFD group was lower than that in the NC group mice, and the ratio will increase after YBT treatment (*p-value <*0.05) **(** **Figure 4A**). The proportion of *Parasutterella* and *Enterococcus* will increase after the inducement of HFFD and decrease after the treatment of YBT (*p-value <*0.05) **(** **Figure 4B**).

In summary, Bacteroidia, Clostridia, Bacilli, Bacteroidia, Clostridia (at the class level), Porphyromonadaceae, Lachnospiraceae (at the family level), and Intestinimonas, Acetatifactor, Alistipes, Parasutterella, and Enterococcus (at the genus level) may not only be the dominant microbiota in the intestines of mice but also the hub associated with YBT in the treatment of NASH.

#### 3.2.4 HFFD and YBT Induced Diversity and Richness Variations in Gut Microbiota

##### 3.2.4.1 Results of Alpha Diversity Analysis

After analyzing, we found that Observed, Chao1, and ACE, which reflect the richness of the gut microbiota, or Shannon and Simpson reflecting the diversity of the microbiota, both have significant differences (*p-value <*0.05) between the NC, HFFD, and HFFD-Y groups.

**Figure 5:**
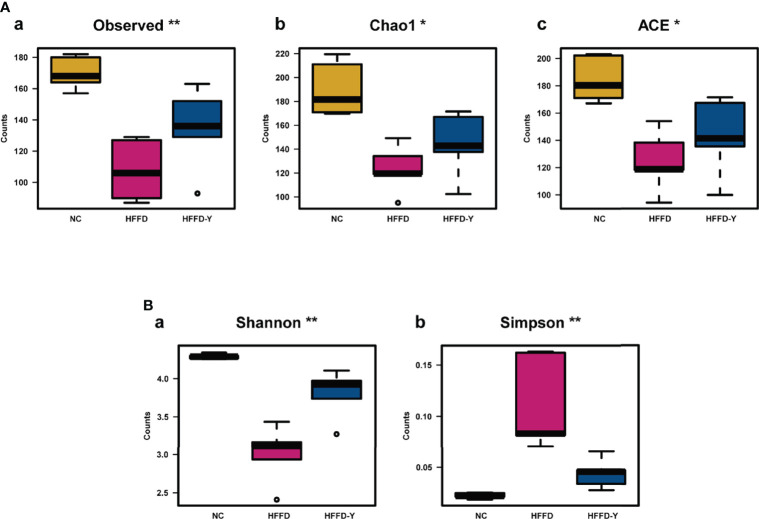
Results of Alpha Diversity analysis. **(A)** Results of the index, reflecting the richness of the gut microbiota. **(B)** Results of the index, reflecting the diversity of the gut microbiota. (*p<0.05 vs HFFD, **p<0.01 vs HFFD).

**Figures 5Aa-c** showed the change in the richness of the mouse gut microbiota. The counts of Observed, Chao1, and ACE both decreased in the HFFD group, compared with the NC group, suggesting the high-fat and high-fructose diet would reduce the microbiota richness of mice. After YBT treatment, the richness of the gut microbiota will be significantly increased (*p-value <*0.05).

In the study, we also noticed a significant increase in Simpson and a decrease in Shannon, indicating the lower microbiota community diversity in the HFFD group compared with the NC group. However, after the YBT intervention, there was a significant decline in Simpson and an increase in Shannon, suggesting an increase in the diversity of microbiota community, compared with the HFFD group (*p-value <*0.05) (**Figures 5Ba, b**).

The research of Astbury S et al. has confirmed that the lower gut microbiome diversity and richness is an actual cause of NASH progress (Astbury et al., 2020). In our study, HFFD-induced NASH mice had significantly lower gut microbiome diversity and richness compared with mice in the NC group. After YBT intervention, the diversity and richness of the gut microbiome were increased considerably, suggesting that YBT possesses the ability to treat NASH mice by improving the diversity and richness of the gut microbiome.

##### 3.2.4.2 Results of Beta Diversity Analysis

The results of β-diversity analysis reflected the similarities and differences of the gut microbiome in each mouse or group.

**Figure 6:**
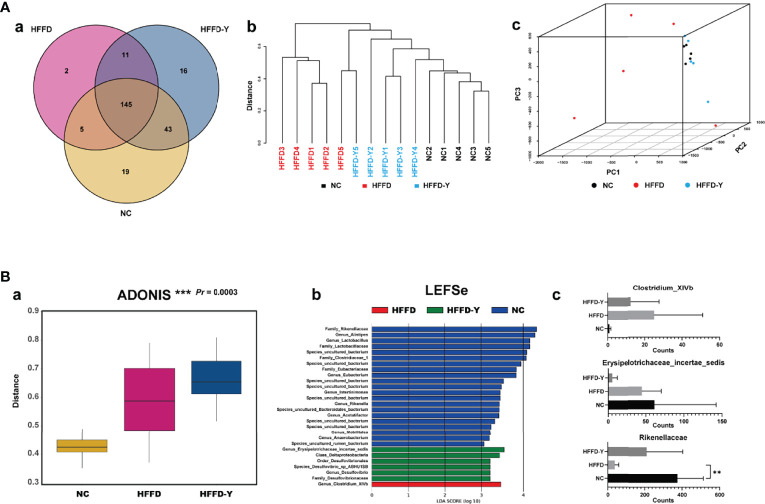
Results of Beta Diversity analysis. **(A)** Results reflect the similarities and differences of the gut microbiome in the mouse. **(B)** Results show the significant difference between the NC, HFFD, HFFD-Y groups, and the gut microbiota, causing the above difference. (**p<0.01 vs HFFD).

The Venn diagram illustrated the exact numbers of OTUs in each group, i.e., 212 OTUs in the NC group, 163 OTUs in the HFFD group, and 215 OTUs in the HFFD-Y group. There were 145 overlapping OTUs in the three groups, which may be the common microbiome of the mice (**Figure 6Aa**). Additionally, each group had its unique OTUs, including 19 OTUs in the NC group, 2 OTUs in the HFFD group, and 16 OTUs in the HFFD-Y group (**Table S1**). Both the clustering tree and PCA results revealed that the clustering results of the NC group and HFFD-Y group were better than those of the HFFD group (the former mice were closer) (**Figures 6Ab-c**). The mice of the NC and HFFD groups are difficult to cluster together, suggesting that the HFFD diet could cause major variations in gut microbiota constitution between the HFFD, NC groups mice. After YBT treatment, the mice of the HFFD-Y group could cluster with mice of the NC group, suggesting that YBT treatment improved the constitution of the gut microbiota and restored it to a similar level to that of NC mice.

ADONIS analysis was used to verify the difference/similarity of gut microbiota between the NC, HFFD, and HFFD-Y groups from statistical. The result showed that the gut microbiota of the three groups of mice is different and statistically significant (*Pr* < 0.05) (**Figure 6Ba**). Next, we used LEFSe analysis to screen out the microbiota that caused the above significant differences between the three groups. The results showed that *Rikenellaceae* (at the level of family), *Erysipelotrichaceae_ incertae_sedis* (at the level of genus), and *Clostridium_XIVb* (at the level of genus) were the most critical microbiota that caused the above-mentioned significant differences in the mice (**Figure 6Bb**). We also screened out the OTUs of *Rikenellaceae* (family), *Erysipelotrichaceae_incertae_sedis* (genus), and *Clostridium_XIVb* (genus), summed the sequencing results of each mouse in the same group to judge the expression changes, and calculated statistical significance. The results showed that the annotation of OTU6, OTU29, OTU35, OTU66, OTU83, OTU90, OTU91, OTU154, OTU174, OTU184, OTU199, and OTU225 are *Rikenellaceae* (family). OTU23, OTU93, OTU119, OTU148, and OTU216 belonged to *Erysipelotrichaceae_incertae_sedis* (genus). OTU37 and OTU43 species annotation results were *Clostridium_XIVb* (genus). Results showed the abundance of *Rikenellaceae* (family) in the mice of the HFFD group was lower than that of NC mice and increased significantly after YBT treatment (*p-value <*0.05). Compared with NC mice, *Clostridium_XIVb* (genus) abundance was raised in the HFFD group and decreased after YBT treatment, but it was not significant (*p-value >*0.05). The counts of *Erysipelotrichaceae_incertae_sedis* (genus) continued to reduce, and YBT did not show an interventional effect on the microbiota (**Figure 6Bc**). The abundance of *Rikenellaceae* (family) after the YBT intervention is significantly increased (*p-value <*0.01), indicating *Rikenellaceae* (family) is the microbiota, associating with the mechanism of YBT treating NASH mice.

#### 3.2.5 PICRUSt Analysis for Predicting the Functional Genomic Changes

Based on the 16S rDNA and reference sequence database, PICRUSt predicted the macrogenome functional composition of the microbiota, with an accuracy of 84-95%.

We screened a total of 82 eligible KEGG pathways, which were used to show the functions of microbiota, between the groups NC and HFFD (**Table S2**). **Table 5** lists the top 15 prediction results with the highest significance (the lowest *p-value*). The results showed that changes in the richness and diversity of the intestinal microbiota between the NC and HFFD groups would induce fatty acids (FAs) metabolism dysfunction, including Arachidonic acid metabolism and Biosynthesis of unsaturated fatty acids. Therefore, we inferred that the HFFD diet-induced decrease in the richness and diversity of gut microbiota and the corresponding fatty acid metabolism disorder is an important pathogenic mechanism of HFFD inducing hepatic steatosis and inflammation in mice.

**Table 5:** PICRUSt predicted results (Top 15) of NC vs. HFFD.

Category	Mean (HFFD)	Mean (NC)	95.0% lower CI	95.0% upper CI	Difference between means	p-value
Translation proteins	0.874890023	0.930738032	-0.072877101	-0.038818918	-0.05584801	6.53E-05
Protein export	0.570890696	0.648995841	-0.104925004	-0.051285285	-0.078105144	0.000150275
Inorganic ion transport and metabolism	0.314941567	0.205608464	0.071004319	0.147661887	0.109333103	0.00017334
Arachidonic acid metabolism	0.027333234	0.009168234	0.011749403	0.024580597	0.018165	0.000182417
Sulfur relay system	0.277160852	0.189979502	0.05603477	0.118327932	0.087181351	0.000197334
Ubiquitin system	0.011082686	0.002531344	0.005394877	0.011707808	0.008551342	0.000246426
Aminoacyl-tRNA biosynthesis	1.042870217	1.210518376	-0.230561541	-0.104734776	-0.167648159	0.000275528
Pyrimidine metabolism	1.698997381	1.981432841	-0.389283154	-0.175587765	-0.28243546	0.000290889
Biosynthesis of unsaturated fatty acids	0.170103188	0.089659632	0.049894747	0.110992365	0.080443556	0.000298439
Caprolactam degradation	0.060707558	0.015310048	0.02814692	0.062648099	0.045397509	0.000299682
Transcription related proteins	0.012791271	0.004541361	0.005114328	0.011385492	0.00824991	0.000300132
Ribosome	2.1258525	2.532428518	-0.562643781	-0.250508255	-0.406576018	0.000320728
Chagas disease (American trypanosomiasis)	0.008481049	1.74E-05	0.005211346	0.0117159	0.008463623	0.000323007
Methane metabolism	1.195459714	1.377981386	-0.254712831	-0.110330513	-0.182521672	0.000391433
African trypanosomiasis	0.009076382	0.001118353	0.004532621	0.011383439	0.00795803	0.000679797

In the HFFD vs. HFFD-Y groups, we found a total of four KEGG pathways (**Table 6**) that met the screening criteria, of which alpha-Linolenic acid (ALA) metabolism had the highest significance (the lowest *p-value*). The most relevant function of the differences in the intestinal microbiota of HFFD and HFFD-Y mice is alpha-Linolenic acid metabolism, suggesting that YBT may achieve the purpose of treating NASH by controlling the regulation of the gut microbiota on the metabolism of ALA. Thus, we believe that YBT could improve the richness and diversity of the intestinal microbiota and the disorder of ALA metabolism of NASH mice. This regulatory mechanism is crucial for YBT to achieve the goal of treating NASH.

**Table 6:** PICRUSt predicted results of HFFD v.s HFFD-Y.

Category	Mean (HFFD)	Mean (NC)	95.0% lower CI	95.0% upper CI	Difference between means	p-value
alpha-Linolenic acid metabolism	0.015560353	0.002938945	0.004363418	0.020879399	0.012621409	0.007794797
Pertussis	0.082692666	0.021797423	0.020738964	0.10105152	0.060895242	0.008115229
Nucleotide excision repair	0.358741223	0.416833767	-0.097146444	-0.019038644	-0.058092544	0.008952555
Synthesis and degradation of ketone bodies	0.02113517	0.030816229	-0.016311732	-0.003050386	-0.009681059	0.009831212

### 3.3 qRT-PCR Results

**Figure 7:**
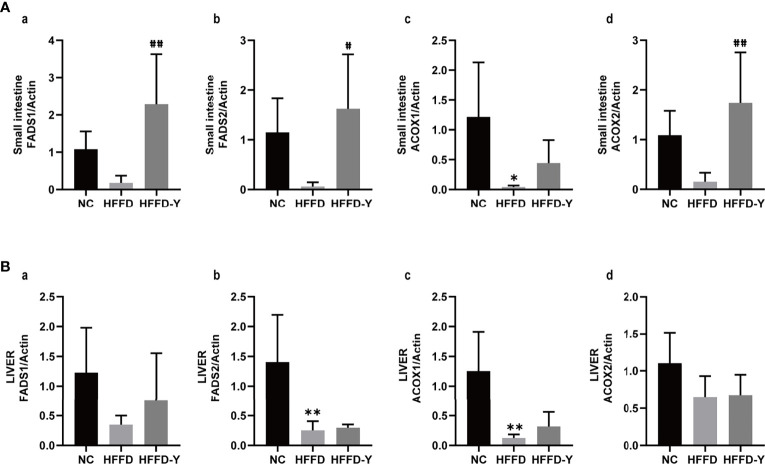
Results of qRT-PCR. **(A)** RNA expression of ALA metabolism-related hub enzymes in the small intestine of mice. **(B)** RNA expression of ALA metabolism-related hub enzymes in the liver of mice. (^*^ *p*<0.05 vs NC, ^**^ *p*<0.01 vs NC; ^#^ *p*<0.05 vs HFFD, ^##^ *p*<0.01 vs HFFD).

The results showed that in all liver and small intestines, the expression of FADS1, FADS2, ACOX1, and ACOX2 were downregulated after the intervention of HFFD. The treatment of YBT will significantly upregulate the expression of the above genes, thereby improving ALA metabolism in mice (**Figure 7**). However, the change of genes in the small intestine of mice was more significant than that in the liver. This difference may suggest the influence of ALA metabolism of YBT is a progressive process. The mechanism of YBT therapy on NASH mice may be related to YBT reshaping the gut microflora distribution of mice and improving ALA metabolism by first increasing the expression of ALA metabolism-related enzymes in the intestinal tract of mice. After that, it acts to restore the expression of these genes in the liver. Further experiments are needed to determine whether this mechanism works through the gut-liver axis.

## 4 Discussion

In the study, we utilized the HFFD diet to induce NASH in mice and administered YBT for treatment to explore the potential treating mechanisms caused by the richness and diversity variations of mice’s gut microbiota. Pathological and biochemical results suggest that YBT may be more effective in treating NASH mice by improving lipid metabolism than improving hepatic inflammation and injury. The result is also consistent with the research team’s finding that YBT has an effect on the treatment of NAFLD/NASH patients, especially for losing weight.

We further investigated the effect of YBT on gut microbiota *via* 16S rDNA analysis and bioinformatics. Compared with the NC group, the richness and diversity of the gut microbiota of mice in the HFFD group were significantly reduced. After YBT intervention, the richness and diversity of microbiota would dramatically be improved. Plaza-Díaz et al. confirmed that the diversity and richness of gut microbiota kept a negative correlation with the deterioration of NAFLD/NASH (Plaza-Díaz et al., 2021). With the complex variety of gut microbiota, a better intestinal protective barrier could be provided, which inhibits the progress of NAFLD/NASH. The clinical research of Pan et al. also determined gut microbiota is a crucial risk factor for Children with NASH (Pan et al., 2021). YBT could indeed achieve the goal of treating NAFLD/NASH by improving the diversity and richness of intestinal microbiota.

The results of LEFSe analysis suggested that *Rikenellaceae* (family), *Erysipelotrichaceae_ incertae_sedis* (genus), and *Clostridium_XIVb* (genus) are the critical microbiota, inducing the above variations of gut microbiota richness and diversity. In addition, we found that *Rikenellaceae* (family) is the target microbiota of YBT, which is related to the acting mechanism of YBT in the treatment of NASH mice. *Rikenellaceae* is a family of bacteria located in the gastrointestinal tract (Rosenberg et al., 2014). Xue et al. confirmed that SI-WU-TANG, another famous TCM in China, could improve the injury of the fibrotic liver by restoring the balance of intestinal microbiota, especially the abundance of *Rikenellaceae* (Xue et al., 2021). High-Fat Diet (HFD) causes a decrease in the *Rikenellaceae* abundance in the intestines of type 2 Diabetes mellitus (T2DM) mice, worsening the condition (Liu et al., 2019). Vallianou et al. further suggested that the decreasing diversity and richness of *Rikenellaceae* is the prime culprit of obesity and metabolism disorder (Vallianou et al., 2021). The high abundance of *Rikenellaceae* is the protective factor of liver and lipid metabolism. It is undoubted that YBT could improve lipid metabolism and treat NASH mice by upregulating the abundance of *Rikenellaceae*.

Considerable studies have revealed that gut microbiota exhibits metabolic function and activity (Cerdó et al., 2019). In the study, the results of PICRUSt analysis and qRT-PCR suggested that YBT could treat NASH mice by improving the richness and diversity of the gut microbiota, which is achieved by improving the function of the microbiota in regulating the metabolism of ALA (an essential omega-3 polyunsaturated fatty acid). ALA is a fatty acid (FA) and a necessary component of cells, primarily located in green plants. Lacking ALA will cause health problems, including metabolic disorders, NAFLD/NASH, and T2DM. Improving ALA metabolism and increasing serum ALA levels help treat metabolism-related diseases. Raspberry seed oil (RO) with a high level of ALA (25.98%) could effectively treat the HFFD-induced NAFLD mice (Hendawy et al., 2021). Han et al. found that the intervention of ALA could activate the AMPK signaling and further improve mitochondrial dysfunction and oxidative stress, treating NAFLD mice induced by HFFD (Han et al., 2021). The high level of ALA in the serum will bring about lower liver inflammation, cancer rate, and balanced lipid metabolism (Wang et al., 2019).

We have identified the regulatory relationship between YBT and intestinal microbiota and the possible mechanisms for subsequent improvement of NASH mice. However, there are still some areas that can be improved. In this study, our research subjects were only mice, and our conclusions (YBT intervention in the intestinal microbiota and subsequent ALA metabolic therapy for NASH) can be further verified by subsequent fecal transplantation in human NAFLD patients. This may be more conducive to the clinical promotion of YBT. In addition, we indirectly reflected the improvement of ALA metabolism in NASH mice by judging the changes in the expression of ALA metabolism-related enzymes. In fact, adding lipid metabolomic analysis in subsequent experiments to visually display the changes of ALA and related metabolites, such as eicosapen taenoicacid (EPA) and docosahexaenoic acid (DHA), is more helpful to confirm the conclusion. In fact, the corresponding experiments are already being designed.

## Conclusion

In summary, we have made it clear that YBT could treat HFFD-induced NASH mice by improving lipid metabolism. The mechanism of YBT to improve lipid metabolism, especially ALA metabolism, is related to the up-regulation of the richness and diversity of mouse intestinal microbiota. Among them, *Rikenellaceae* is a critical bacterial. We believe that the results of the study can provide theoretical support for the clinical promotion of YBT.

## Data Availability Statement

The original contributions presented in the study are publicly available. This data can be found here: https://ngdc.cncb.ac.cn/gsa/, CRA005563.

## Ethics Statement

The animal study was reviewed and approved by The Animal Ethics Committee of Zhejiang Chinese Medical University.

## Author Contributions

BH and ZC were responsible for providing ideas for the study. JY and YN were the principal actors in the study, including data analyses and manuscript writing. JL, YL, and LW were mainly responsible for model construction and pathological data analysis. All authors approved the manuscript.

## Funding

This study was supported by research grants from the Zhejiang Provincial Natural Science Foundation of China (Grant No.LGF22H290001, LY17H290007, LQ20H290002), National Natural Science Foundation of China (82004262), the Zhejiang Traditional Chinese Medicine Administration (CN) (Grant No.2020ZB081), the Health Commission of Zhejiang province (Grant No.2018KY550), and the Zhejiang Provincial Key Lab of Diagnosis and Treatment of Circulatory Diseases (Grant No. 2019E10012).

## Conflict of Interest

The authors declare that the research was conducted in the absence of any commercial or financial relationships that could be construed as a potential conflict of interest.

## Publisher’s Note

All claims expressed in this article are solely those of the authors and do not necessarily represent those of their affiliated organizations, or those of the publisher, the editors and the reviewers. Any product that may be evaluated in this article, or claim that may be made by its manufacturer, is not guaranteed or endorsed by the publisher.

## Supplementary Material

Supplementary Figure 1 The evolutionary tree of top 100 OTUs.

## Abbreviations

ACOX1, Acyl-CoA Oxidase 1; ACOX2, Acyl-CoA Oxidase 2;ALA, α-Linolenic acid; ANOVA, One-way analysis of variance; CVDs, Cardiovascular diseases; DHA, Docosahexaenoic acid; EPA, Eicosapen taenoicacid; FADS1, Fatty acid desaturase 1; FADS2, Fatty acid desaturase 2; HCC, Hepatocellular carcinoma; HDL-c, High-density lipoprotein cholesterol; H&E staining, Hematoxylin and Eosin staining; HFFD, High-fat and high-fructose diet; LDL-c, Low-density lipoprotein cholesterol; MS, Metabolic syndrome; NAFLD, Nonalcoholic fatty liver disease; NAS, NAFLD activity score; NASH, Nonalcoholic steatohepatitis; OTU, Operational Taxonomic Unit; PUFAs, Polyunsaturated fatty acids; RO, Raspberry seed oil; SEM, Standard error of mean; T2DM, Type 2 Diabetes mellitus; UHPLC-MS/MS, Ultra-high performance liquid chromatography-MS/MS; YBT, Yiqi-Bushen-Tiaozhi.
