# Supplementary figures and images for: Yiqi-Bushen-Tiaozhi Recipe Attenuated High-Fat and High-Fructose Diet Induced Nonalcoholic Steatohepatitis in Mice via Gut Microbiota
Source: Front Cell Infect Microbiol. 2022 Apr 22;12:824597. doi: 10.3389/fcimb.2022.824597 (PMC9072834; doi:10.3389/fcimb.2022.824597)

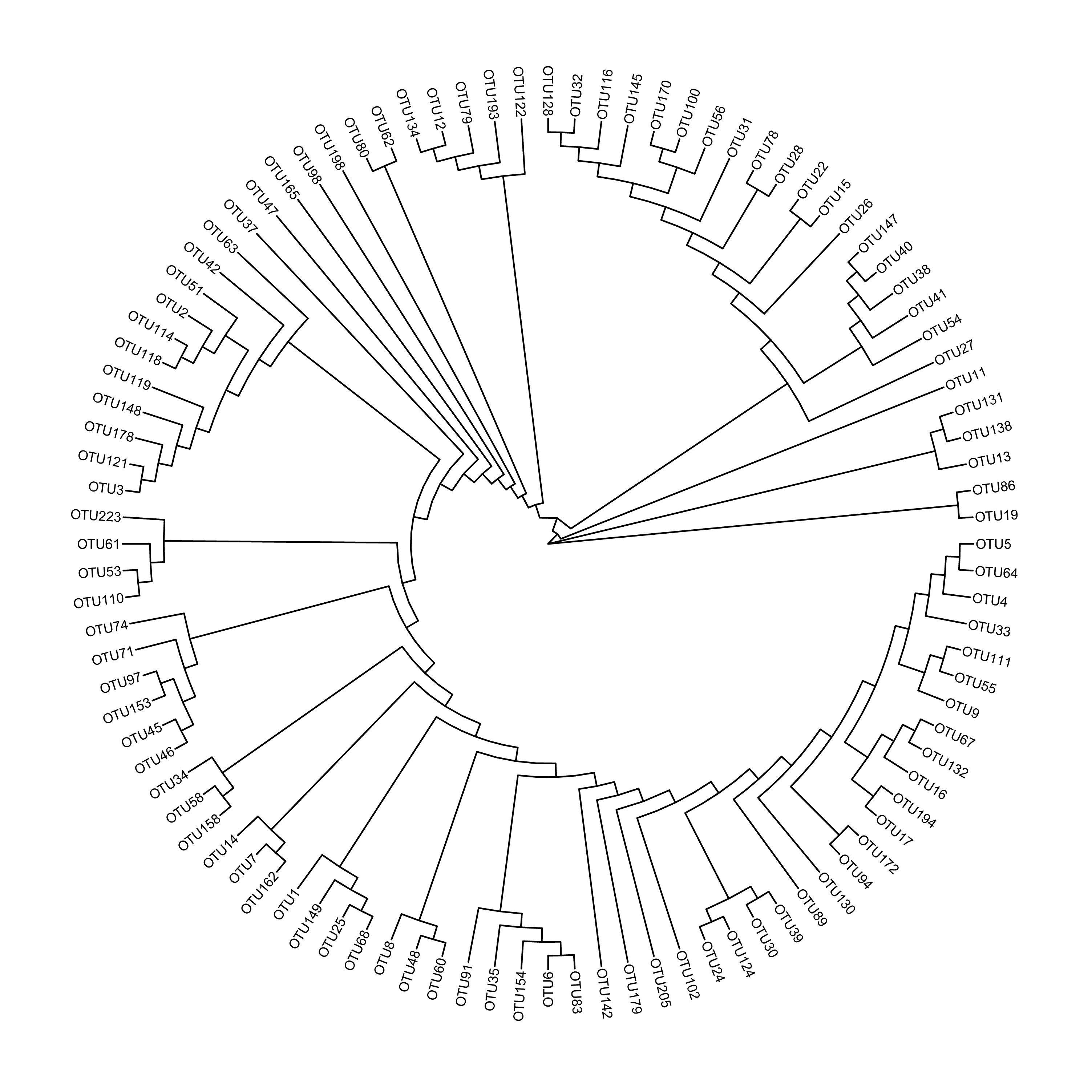

Supplement: Supplementary Figure 1 — The evolutionary tree of top 100 OTUs. [file Image_1.tif]
