# Supplementary material for: Yiqi-Bushen-Tiaozhi Recipe Attenuated High-Fat and High-Fructose Diet Induced Nonalcoholic Steatohepatitis in Mice via Gut Microbiota
Source: Front Cell Infect Microbiol. 2022 Apr 22;12:824597. doi: 10.3389/fcimb.2022.824597 (PMC9072834; doi:10.3389/fcimb.2022.824597)
Supplement: Supplementary file 2 [file Table_1.docx]

**Table S1.** The unique OTUs of NC, HFFD, and HFFD-Y groups

| **ID** | **Superkingdom** | **Phylum** | **Class** | **Order** | **Family** | **Genus** | **Species** |
| --- | --- | --- | --- | --- | --- | --- | --- |
| **NC group** | | | | | | | |
| OTU212 | Bacteria | Actinobacteria | Actinobacteria | Coriobacteriales | Coriobacteriaceae |  | uncultured_bacterium |
| OTU214 | Bacteria | Firmicutes |  |  |  |  | uncultured_rumen_bacterium |
| OTU217 | Bacteria | Firmicutes | Clostridia | Clostridiales | Ruminococcaceae | Anaerobacterium | uncultured_bacterium |
| OTU218 | Bacteria | Proteobacteria | Alphaproteobacteria | Rhodospirillales | Rhodospirillaceae | Aestuariispira | uncultured_rumen_bacterium |
| OTU219 | Bacteria | Firmicutes | Clostridia | Clostridiales | Ruminococcaceae | Unassigned | Unassigned |
| OTU222 | Bacteria | Firmicutes | Clostridia | Clostridiales | Ruminococcaceae |  | Unassigned |
| OTU227 | Bacteria | Firmicutes | Clostridia | Clostridiales | Ruminococcaceae | Unassigned | Unassigned |
| OTU230 | Bacteria | Firmicutes | Erysipelotrichia | Erysipelotrichales | Erysipelotrichaceae | Allobaculum | uncultured_organism |
| OTU231 | Bacteria | Firmicutes | Clostridia | Clostridiales | Ruminococcaceae | Anaerobacterium | Unassigned |
| OTU232 | Bacteria | Firmicutes | Clostridia | Clostridiales | Ruminococcaceae |  | Unassigned |
| OTU233 | Bacteria | Firmicutes | Clostridia | Clostridiales | Lachnospiraceae | Mobilitalea | uncultured_bacterium |
| OTU234 | Bacteria | Firmicutes | Clostridia | Clostridiales | Lachnospiraceae | Lachnospiracea_incertae_sedis | Unassigned |
| OTU235 | Bacteria | Firmicutes | Clostridia | Clostridiales | Lachnospiraceae | Mobilitalea | uncultured_bacterium |
| OTU236 | Bacteria | Firmicutes | Clostridia | Clostridiales | Lachnospiraceae |  | Unassigned |
| OTU237 | Bacteria | Firmicutes | Clostridia | Clostridiales | Ruminococcaceae | Unassigned | Unassigned |
| OTU238 | Bacteria | Firmicutes | Clostridia | Clostridiales | Lachnospiraceae | Unassigned | Unassigned |
| OTU239 | Bacteria | Firmicutes | Clostridia | Clostridiales | Unassigned | Unassigned | Unassigned |
| OTU240 | Bacteria | Unassigned | Unassigned | Unassigned | Unassigned | Unassigned | Unassigned |
| OTU241 | Bacteria | Firmicutes | Unassigned | Unassigned | Unassigned | Unassigned | Unassigned |
| **HFFD group** | | | | | | | |
| OTU157 | Bacteria | Firmicutes | Clostridia | Clostridiales | Ruminococcaceae | Unassigned | Unassigned |
| OTU169 | Bacteria | Proteobacteria | Deltaproteobacteria | Desulfovibrionales | Desulfovibrionaceae | Desulfovibrio | Desulfovibrio_sp._ABHU1SB |
| OTU173 | Bacteria | Firmicutes | Clostridia | Clostridiales | Ruminococcaceae | Unassigned | Unassigned |
| OTU179 | Bacteria | Bacteroidetes | Bacteroidia | Bacteroidales | Porphyromonadaceae | Odoribacter | uncultured_bacterium |
| OTU189 | Bacteria | Firmicutes | Clostridia | Clostridiales | Ruminococcaceae | Unassigned | Unassigned |
| OTU191 | Bacteria | Bacteroidetes | Bacteroidia | Bacteroidales | Porphyromonadaceae |  | uncultured_bacterium |
| OTU197 | Bacteria | Bacteroidetes | Bacteroidia | Bacteroidales | Prevotellaceae | Prevotella | Unassigned |
| OTU199 | Bacteria | Bacteroidetes | Bacteroidia | Bacteroidales | Rikenellaceae | Rikenella | Unassigned |
| OTU200 | Bacteria | Firmicutes | Clostridia | Clostridiales |  |  | Unassigned |
| OTU204 | Bacteria | Firmicutes | Clostridia | Clostridiales | Ruminococcaceae |  | Unassigned |
| OTU206 | Bacteria | Bacteroidetes | Bacteroidia | Bacteroidales | Prevotellaceae | Alloprevotella | uncultured_bacterium |
| OTU207 | Bacteria | Firmicutes | Bacilli | Lactobacillales | Streptococcaceae | Lactococcus | Unassigned |
| OTU210 | Bacteria | Proteobacteria | Alphaproteobacteria | Rhodospirillales | Rhodospirillaceae | Aestuariispira | Unassigned |
| OTU211 | Bacteria | Bacteroidetes | Unassigned | Unassigned | Unassigned | Unassigned | Unassigned |
| OTU213 | Bacteria | Actinobacteria | Actinobacteria | Bifidobacteriales | Bifidobacteriaceae | Bifidobacterium | Unassigned |
| OTU226 | Bacteria | Firmicutes | Erysipelotrichia | Erysipelotrichales | Erysipelotrichaceae | Clostridium_XVIII | Clostridium_ramosum |
| **HFFD-Y group** | | | | | | | |
| OTU14 | Bacteria | Proteobacteria | Betaproteobacteria | Burkholderiales | Sutterellaceae | Parasutterella | Unassigned |
| OTU113 | Bacteria | Firmicutes | Clostridia | Clostridiales | Ruminococcaceae | Unassigned | Unassigned |
