# Supplementary material for: Yiqi-Bushen-Tiaozhi Recipe Attenuated High-Fat and High-Fructose Diet Induced Nonalcoholic Steatohepatitis in Mice via Gut Microbiota
Source: Front Cell Infect Microbiol. 2022 Apr 22;12:824597. doi: 10.3389/fcimb.2022.824597 (PMC9072834; doi:10.3389/fcimb.2022.824597)
Supplement: Supplementary file 3 [file Table_2.docx]

**Table S2.** PICRUSt predicted results of NC v.s HFFD

| **Category** | **Mean (HFFD)** | **Mean (NC)** | **95.0% lower CI** | **95.0% upper CI** | **Difference between means** | **p-value** |
| --- | --- | --- | --- | --- | --- | --- |
| Translation proteins | 0.874890023 | 0.930738032 | -0.072877101 | -0.038818918 | -0.05584801 | 6.53E-05 |
| Protein export | 0.570890696 | 0.648995841 | -0.104925004 | -0.051285285 | -0.078105144 | 0.000150275 |
| Inorganic ion transport and metabolism | 0.314941567 | 0.205608464 | 0.071004319 | 0.147661887 | 0.109333103 | 0.00017334 |
| Arachidonic acid metabolism | 0.027333234 | 0.009168234 | 0.011749403 | 0.024580597 | 0.018165 | 0.000182417 |
| Sulfur relay system | 0.277160852 | 0.189979502 | 0.05603477 | 0.118327932 | 0.087181351 | 0.000197334 |
| Ubiquitin system | 0.011082686 | 0.002531344 | 0.005394877 | 0.011707808 | 0.008551342 | 0.000246426 |
| Aminoacyl-tRNA biosynthesis | 1.042870217 | 1.210518376 | -0.230561541 | -0.104734776 | -0.167648159 | 0.000275528 |
| Pyrimidine metabolism | 1.698997381 | 1.981432841 | -0.389283154 | -0.175587765 | -0.28243546 | 0.000290889 |
| Biosynthesis of unsaturated fatty acids | 0.170103188 | 0.089659632 | 0.049894747 | 0.110992365 | 0.080443556 | 0.000298439 |
| Caprolactam degradation | 0.060707558 | 0.015310048 | 0.02814692 | 0.062648099 | 0.045397509 | 0.000299682 |
| Transcription related proteins | 0.012791271 | 0.004541361 | 0.005114328 | 0.011385492 | 0.00824991 | 0.000300132 |
| Ribosome | 2.1258525 | 2.532428518 | -0.562643781 | -0.250508255 | -0.406576018 | 0.000320728 |
| Chagas disease (American trypanosomiasis) | 0.008481049 | 1.74E-05 | 0.005211346 | 0.0117159 | 0.008463623 | 0.000323007 |
| Methane metabolism | 1.195459714 | 1.377981386 | -0.254712831 | -0.110330513 | -0.182521672 | 0.000391433 |
| African trypanosomiasis | 0.009076382 | 0.001118353 | 0.004532621 | 0.011383439 | 0.00795803 | 0.000679797 |
| Geraniol degradation | 0.086176998 | 0.040392084 | 0.025924628 | 0.065645199 | 0.045784913 | 0.000714343 |
| Homologous recombination | 0.862108512 | 0.986920439 | -0.179160948 | -0.070462905 | -0.124811926 | 0.000732169 |
| Translation factors | 0.518141899 | 0.593275806 | -0.107962606 | -0.042305207 | -0.075133907 | 0.000748312 |
| Glyoxylate and dicarboxylate metabolism | 0.572993869 | 0.521319333 | 0.029094972 | 0.074254101 | 0.051674537 | 0.000748546 |
| Thiamine metabolism | 0.461033753 | 0.510872106 | -0.072039327 | -0.027637378 | -0.049838352 | 0.000846181 |
| Biosynthesis and biodegradation of secondary metabolites | 0.078061276 | 0.059413506 | 0.010289705 | 0.027005835 | 0.01864777 | 0.000879763 |
| Phenylalanine metabolism | 0.222391339 | 0.180648373 | 0.022999889 | 0.060486043 | 0.041742966 | 0.000889788 |
| Lysine biosynthesis | 0.763831551 | 0.851382302 | -0.127030552 | -0.048070952 | -0.087550752 | 0.000914132 |
| Drug metabolism - cytochrome P450 | 0.049085085 | 0.018730632 | 0.016501376 | 0.044207531 | 0.030354454 | 0.000985761 |
| Fatty acid biosynthesis | 0.473301375 | 0.439003369 | 0.018572546 | 0.050023467 | 0.034298006 | 0.001014785 |
| Glutathione metabolism | 0.230698794 | 0.166112919 | 0.034793273 | 0.094378476 | 0.064585875 | 0.00105405 |
| Basal transcription factors | 8.02E-06 | 0.000319095 | -0.000455999 | -0.000166151 | -0.000311075 | 0.001121193 |
| Bacterial secretion system | 0.719764755 | 0.578314396 | 0.075381296 | 0.207519422 | 0.141450359 | 0.001139309 |
| Metabolism of cofactors and vitamins | 0.128872769 | 0.087463533 | 0.021920994 | 0.06089748 | 0.041409237 | 0.001193965 |
| Replication, recombination and repair proteins | 0.893727473 | 0.674548902 | 0.115912802 | 0.32244434 | 0.219178571 | 0.00120221 |
| Others | 1.105185463 | 0.91607548 | 0.099922333 | 0.278297635 | 0.189109984 | 0.001209631 |
| Drug metabolism - other enzymes | 0.278118957 | 0.351266221 | -0.10781407 | -0.038480458 | -0.073147264 | 0.00124677 |
| Signal transduction mechanisms | 0.483658582 | 0.384267572 | 0.051798024 | 0.146983997 | 0.099391011 | 0.001328541 |
| Amino acid related enzymes | 1.385482622 | 1.538252022 | -0.226006525 | -0.079532276 | -0.1527694 | 0.001337952 |
| Peptidoglycan biosynthesis | 0.753857999 | 0.815440705 | -0.091257505 | -0.031907908 | -0.061582706 | 0.001380839 |
| Lysine degradation | 0.162778227 | 0.119423731 | 0.022405992 | 0.064302998 | 0.043354495 | 0.001404196 |
| Tetracycline biosynthesis | 0.134791891 | 0.100927941 | 0.017489613 | 0.050238287 | 0.03386395 | 0.001410287 |
| Biosynthesis of siderophore group nonribosomal peptides | 0.033550461 | 0.007790882 | 0.012984083 | 0.038535074 | 0.025759579 | 0.001645409 |
| Cell cycle - Caulobacter | 0.471812041 | 0.545192658 | -0.109822527 | -0.036938708 | -0.073380617 | 0.001658759 |
| Glycosyltransferases | 0.402355803 | 0.320005749 | 0.041232558 | 0.123467551 | 0.082350054 | 0.001713636 |
| DNA replication | 0.637647106 | 0.713049284 | -0.113214113 | -0.037590242 | -0.075402178 | 0.001758926 |
| Chromosome | 1.477663031 | 1.59090227 | -0.170164089 | -0.056314389 | -0.113239239 | 0.001784912 |
| Other ion-coupled transporters | 1.392530339 | 1.189637704 | 0.100028072 | 0.305757196 | 0.202892634 | 0.001878273 |
| Retinol metabolism | 0.034556867 | 0.018871503 | 0.007699041 | 0.023671689 | 0.015685365 | 0.001926762 |
| Function unknown | 1.542947506 | 1.281946131 | 0.127655282 | 0.394347467 | 0.261001375 | 0.001966457 |
| D-Glutamine and D-glutamate metabolism | 0.140905473 | 0.168289379 | -0.041398521 | -0.013369291 | -0.027383906 | 0.001986739 |
| Mismatch repair | 0.759748353 | 0.852458496 | -0.140765116 | -0.044655169 | -0.092710143 | 0.002142538 |
| DNA replication proteins | 1.18299583 | 1.319339464 | -0.207084704 | -0.065602565 | -0.136343634 | 0.002154992 |
| Type I diabetes mellitus | 0.057795944 | 0.070997445 | -0.020101579 | -0.006301423 | -0.013201501 | 0.002250575 |
| Energy metabolism | 0.928093511 | 1.030804896 | -0.158334975 | -0.047087796 | -0.102711385 | 0.002768383 |
| Epithelial cell signaling in Helicobacter pylori infection | 0.077133252 | 0.092302068 | -0.023439539 | -0.006898094 | -0.015168816 | 0.00287911 |
| Cytoskeleton proteins | 0.272478471 | 0.338369994 | -0.102109878 | -0.02967317 | -0.065891524 | 0.003015907 |
| Phosphatidylinositol signaling system | 0.111931828 | 0.092626657 | 0.008658269 | 0.029952072 | 0.019305171 | 0.003074176 |
| Terpenoid backbone biosynthesis | 0.517047966 | 0.592935366 | -0.117823218 | -0.033951583 | -0.0758874 | 0.003109443 |
| Fluorobenzoate degradation | 0.01252373 | 5.77E-05 | 0.005522579 | 0.0194094 | 0.01246599 | 0.003252801 |
| RNA polymerase | 0.149214286 | 0.164490193 | -0.023806035 | -0.00674578 | -0.015275908 | 0.003300118 |
| One carbon pool by folate | 0.608457724 | 0.707761134 | -0.154781879 | -0.043824941 | -0.09930341 | 0.00330928 |
| Renal cell carcinoma | 0.012608309 | 0.004787013 | 0.003402936 | 0.012239656 | 0.007821296 | 0.003524161 |
| Photosynthesis proteins | 0.343082835 | 0.383913921 | -0.064181768 | -0.017480404 | -0.040831086 | 0.003776024 |
| Metabolism of xenobiotics by cytochrome P450 | 0.045057181 | 0.018715902 | 0.011269999 | 0.041412558 | 0.026341279 | 0.003786015 |
| Other transporters | 0.296537709 | 0.237508836 | 0.024978225 | 0.09307952 | 0.059028873 | 0.003963034 |
| Glycerophospholipid metabolism | 0.573539582 | 0.538352923 | 0.014773108 | 0.05560021 | 0.035186659 | 0.004091127 |
| Tyrosine metabolism | 0.377947005 | 0.313027821 | 0.027049667 | 0.102788701 | 0.064919184 | 0.004217338 |
| Plant-pathogen interaction | 0.136770743 | 0.164031438 | -0.04356216 | -0.010959232 | -0.027260696 | 0.004834211 |
| C5-Branched dibasic acid metabolism | 0.283924037 | 0.308792703 | -0.039773549 | -0.009963784 | -0.024868666 | 0.004894515 |
| Limonene and pinene degradation | 0.128546679 | 0.097414312 | 0.012245358 | 0.050019377 | 0.031132367 | 0.005228481 |
| Photosynthesis | 0.334003566 | 0.377613139 | -0.070162693 | -0.017056452 | -0.043609573 | 0.005332543 |
| Peptidases | 1.817258923 | 1.94474254 | -0.206445563 | -0.04852167 | -0.127483617 | 0.005845912 |
| Fatty acid metabolism | 0.263044285 | 0.201506904 | 0.023326943 | 0.099747818 | 0.061537381 | 0.005924018 |
| Fructose and mannose metabolism | 0.969650288 | 0.882747101 | 0.032576815 | 0.141229559 | 0.086903187 | 0.006140807 |
| Huntington's disease | 0.050615385 | 0.039451473 | 0.004142162 | 0.018185662 | 0.011163912 | 0.006343016 |
| Ribosome Biogenesis | 1.354682161 | 1.43082054 | -0.124732932 | -0.027543826 | -0.076138379 | 0.006851117 |
| Ubiquinone and other terpenoid-quinone biosynthesis | 0.298849445 | 0.230213188 | 0.024827608 | 0.112444906 | 0.068636257 | 0.006852961 |
| DNA repair and recombination proteins | 2.725607993 | 2.890276071 | -0.270073432 | -0.059262724 | -0.164668078 | 0.006956759 |
| Phenylalanine, tyrosine and tryptophan biosynthesis | 0.776187104 | 0.861009416 | -0.139817018 | -0.029827606 | -0.084822312 | 0.007436228 |
| Secretion system | 1.445346925 | 1.080711152 | 0.124072731 | 0.605198814 | 0.364635773 | 0.008134365 |
| D-Arginine and D-ornithine metabolism | 0.002344333 | 0.004720304 | -0.003964389 | -0.000787553 | -0.002375971 | 0.008703216 |
| General function prediction only | 3.670972854 | 3.571317104 | 0.032667342 | 0.166644159 | 0.09965575 | 0.008947685 |
| Pantothenate and CoA biosynthesis | 0.579017359 | 0.647050028 | -0.114072033 | -0.021993305 | -0.068032669 | 0.009255941 |
| Protein folding and associated processing | 0.748768206 | 0.680809319 | 0.021556992 | 0.114360781 | 0.067958887 | 0.009680183 |
| Lipopolysaccharide biosynthesis proteins | 0.601821736 | 0.452799956 | 0.046629018 | 0.251414542 | 0.14902178 | 0.009988778 |
| Naphthalene degradation | 0.162462937 | 0.137841332 | 0.007702142 | 0.041541068 | 0.024621605 | 0.009994587 |
